# ASPHER statement on racism and health: racism and discrimination obstruct public health’s pursuit of health equity

**DOI:** 10.1007/s00038-020-01442-y

**Published:** 2020-07-18

**Authors:** Nurcan Akbulut, Naomi Limaro, Lisa Wandschneider, Rhanjeet Dhonkal, Nadav Davidovitch, John Middleton, Oliver Razum

**Affiliations:** 1 Bielefeld School of Public Health, Bielefeld University, Bielefeld, Germany; 2 ASPHER, Brussels, Belgium; 3 HAW Hamburg, Hamburg, Germany; 4 School of Public Health, Ben-Gurion University of the Negev, Be’er Sheva, Israel

The COVID-19 pandemic has unmasked structural racial inequalities. Association of Schools of Public Health in the European Region (ASPHER) member schools need to act against racism now.

The COVID-19 public health crisis has elicited strong public health system responses. But the pandemic has also uncovered profound and neglected structural inequalities and injustices in our societies.

These structural inequalities developed through enduring discrimination against ethnic, cultural and other minority groups. They became apparent in several ways over the last 6 months.

- People of Asian descent experienced discrimination in public spaces in reaction to the pandemic presumably having originated in China (Devakumar et al. 2020a).
- Ethnic/racial minority groups in Europe are more adversely affected by the COVID-19 pandemic compared to most white people. In the UK, black people are four times more likely to die from COVID-19 than white people, even after controlling for socio-economic disadvantage (Platt and Warwick 2020).
- Ethnic/racial minority groups in Europe often live in crowded conditions, especially so when they are refugees. Under such circumstances, physical distancing is a privilege that they cannot afford (Bozorgmehr et al. 2020).
- Ethnic/racial minority groups in Europe often live in poor social conditions with precarious forms of employment, so they suffer most from the adverse socio-economic consequences of the pandemic. At the same time, they lack equal access to health care as well as social protection, putting them at greater risk of adverse health outcomes.
- Ethnic/racial minority groups in Europe are also often in occupations which have key functions in the pandemic. Examples are health and social care, transport, delivery services, food supply and security roles. Workers in these fields have been particularly vulnerable to infection (Devakumar et al. 2020a).

In summary, the pandemic has not only caused a global public health crisis; it has also increased and accentuated longstanding structural social inequalities and ethnic/racial discrimination (Devakumar et al. 2020b).

The amalgamation of different forms of inequalities resulting from racism and socio-economic disadvantages signals an urgent need to protect the health of vulnerable groups. On the one hand, social inequalities which the pandemic reinforces need to be tackled; on the other hand, inappropriate government policy responses to it must be addressed. A striking current instance of this fact is provided in the failure of the European Union and its member states to evacuate migrants and refugees from the camps on Greek islands to enable living circumstances that allow physical distancing and provide safe spaces (Veizis 2020). Apart from the UK rapid review, there is as yet little work addressing the differential ethnic/racial impact of the pandemic or of social countermeasures taken, of diminished health and social care and of economic disruption. ASPHER, as Europe’s representative organization for Schools of Public Health, accordingly has issued its first statement on COVID-19 impact on Health Inequalities and Vulnerable Populations on 2 June 2020 (ASPHER 2020). In addition, ASPHER will pursue health equity by fighting systemic racism and discrimination.

1. We will continue to call upon all public health organizations and governments in all countries to strengthen the protection of the health of vulnerable groups. We also call for urgent and decisive action to minimize the social impact of the COVID-19 pandemic on socially and economically marginalized minorities.
2. Racism has a considerable impact on health inequalities. We therefore call on public health scientists to routinely include racism as a fundamental social determinant of health in all research and to strengthen cross-disciplinary collaboration on issues related to racism and health.
3. We need to rigorously name and scrutinize such systemic disadvantages for what they are, i.e. structural racism (Hardeman et al. 2018). It is a task for society to put an end to systemic racism and structural inequalities through civic engagement, critical awareness, education, equal opportunities in life, political integrity and scientific evidence. In addition, we must hold politicians accountable for their actions, including their handling of information and media.
4. We advocate for communities and governments to embrace comprehensive public health strategies for addressing all causes of violence in our cities and places. Preventive models addressing communities as a whole have to be implemented to address violence and inequalities. These must include significant partnership working and retraining of all statutory workers including those with regulatory powers and workforces, including police, prisons and places of detention.
5. ASPHER member schools of public health should be role models for eliminating all forms of racism, discrimination, inequality and disadvantage.
6. We reassert our commitment to health as a fundamental human right, to equality and fairness, to respect for all people worldwide, to solidarity with oppressed people and to protecting and improving the health of all the people we serve. Member schools should review systematically their curricula and teaching with respect to racism, discrimination and inequalities in health and in public health interventions to reduce inequalities and improve health more fairly.
7. We also call on schools of public health to critically address their own policies with regard to racism and discrimination—as employers, and in their recruitment of staff and students; as landowners and procurers of goods and services, and in their policies towards acceptance of grants and donations.
8. We call on all our schools of public health to work within their academic institutions to audit, review and develop policies and programmes to address and eliminate racism.

Racism and discrimination are public health issues, globally and in Europe. They are contributing factors to the COVID-19 crisis. As public health researchers and practitioners, we must be aware of this. We need to take the necessary actions to address racism and discrimination in order to attain health equity.

## References

[CR1] ASPHER (2020) COVID-19—How and why is the pandemic exacerbating and amplifying health inequalities and vulnerabilities in Europe? ASPHER, Brussels. https://www.aspher.org/download/428/aspher-covid-19-task-force-first-statement-on-health-inequalities-and-vulnerable-populations.pdf. Accessed 19 June 2020

[CR2] Bozorgmehr K, Hintermeier M, Razum O et al (2020) SARS‐CoV‐2 in Aufnahmeeinrichtungen und Gemeinschaftsunterkünften für Geflüchtete: epidemiologische und normativ‐rechtliche Aspekte (V. 1, 29. Mai 2020 ed.). Bremen: Kompetenznetz Public Health COVID-19. 10.4119/unibi/2943665. Accessed 19 June 2020 (**in German**)

[CR3] Devakumar D, Shannon G, Bhopal SS, Abubakar I (2020a) Racism and discrimination in COVID-19 responses. Lancet 395(10231):1194. https://www.thelancet.com/pdfs/journals/lancet/PIIS0140-6736(20)30792-3.pdf. Accessed 26 June 2020 10.1016/S0140-6736(20)30792-3 PMC7146645 32246915

[CR4] Devakumar D, Selvarajah S, Shannon G et al (2020b). Racism, the public health crisis we can no longer ignore. Lancet. https://www.thelancet.com/pdfs/journals/lancet/PIIS0140-6736(20)31371-4.pdf. Accessed 26 June 2020 10.1016/S0140-6736(20)31371-4 PMC7289562 32534630

[CR5] Hardeman RR, Murphy KA, Karbeah JM, Kozhimannil KB (2018). Naming institutionalized racism in the public health literature: a systematic literature review. Public Health Rep.

[CR6] Platt L, Warwick R (2020) Are some ethnic groups more vulnerable to COVID-19 than others? The Institute for Fiscal Studies, London. https://www.ifs.org.uk/inequality/wp-content/uploads/2020/04/Are-some-ethnic-groups-more-vulnerable-to-COVID-19-than-others-V2-IFS-Briefing-Note.pdf. Accessed 26 June 2020

[CR7] Veizis A (2020) Commentary: “Leave No One Behind” and access to protection in the Greek islands in the COVID-19 Era. Int Migr 58(3):264–266. https://doi.org/10.1111/imig.12721. Accessed 26 June 2020 10.1111/imig.12721 PMC7272862 32518422

